# SARS-CoV2 mRNA Vaccine-Specific B-, T- and Humoral Responses in Adolescents After Kidney Transplantation

**DOI:** 10.3389/ti.2022.10677

**Published:** 2022-08-04

**Authors:** Arne Sattler, Julia Thumfart, Laura Tóth, Eva Schrezenmeier, Vanessa Proß, Carolin Stahl, Janine Siegle, An He, Linda Marie Laura Thole, Carolin Ludwig, Henriette Straub-Hohenbleicher, Frank Friedersdorff, Bernd Jahrsdörfer, Hubert Schrezenmeier, Philip Bufler, Katja Kotsch

**Affiliations:** ^1^ Department of General and Visceral Surgery, Charite-Universitätsmedizin Berlin, Corporate Member of Freie Universität Berlin and Humboldt-Universität zu Berlin, Berlin, Germany; ^2^ Department of Pediatric Gastroenterology, Nephrology and Metabolic Diseases, Charité-Universitätsmedizin Berlin, Corporate Member of Freie Universität Berlin and Humboldt-Universität zu Berlin, Berlin, Germany; ^3^ Department of Nephrology and Medical Intensive Care, Charité-Universitätsmedizin Berlin, Corporate Member of Freie Universität Berlin and Humboldt-Universität zu Berlin, Berlin, Germany; ^4^ Department of Rheumatology and Clinical Immunology, Charité-Universitätsmedizin Berlin, Corporate Member of Freie Universität Berlin and Humboldt-Universität zu Berlin, Berlin, Germany; ^5^ Berlin Institute of Health at Charité-Universitätsmedizin Berlin, BIH Academy, Clinician Scientist Program Universitätsmedizin Berlin, Berlin, Germany; ^6^ Department of Urology, Evangelisches Krankenhaus Königin Elisabeth Herzberge, Berlin, Germany; ^7^ Department of Urology, Charite-Universitätsmedizin Berlin, Corporate Member of Freie Universität Berlin and Humboldt-Universität zu Berlin, Berlin, Germany; ^8^ Institute of Transfusion Medicine, Ulm University, Ulm, Germany; ^9^ Institute for Clinical Transfusion Medicine and Immunogenetics, German Red Cross Blood Transfusion Service Baden-Württemberg—Hessen and University Hospital Ulm, Ulm, Germany

**Keywords:** kidney transplantation, vaccination, immunity, SARS-CoV2, adolescents

## Abstract

Protection of adult kidney transplant recipients against SARS-CoV2 was shown to be strongly impaired owing to low reactogenicity of available vaccines. So far, data on vaccination outcomes in adolescents are scarce due to later vaccination approval for this age group. We therefore comprehensively analyzed vaccination-specific humoral-, T- and B-cell responses in kidney transplanted adolescents aged 12–18 years in comparison to healthy controls 6 weeks after standard two-dose BNT162b2 (“Comirnaty”; Pfizer/BioNTech) vaccination. Importantly, 90% (18/20) of transplanted adolescents showed IgG seroconversion with 75% (15/20) developing neutralizing titers. Still, both features were significantly diminished in magnitude compared to controls. Correspondingly, spike-specific B cells were quantitatively reduced and enriched for non-isotype-class-switched IgD^+^27^+^ memory cells in patients. Whereas spike specific CD4^+^ T cell frequencies were similar in both groups, cytokine production and memory differentiation were significantly impaired in transplant recipients. Although our data identify limitations in all arms of vaccine-specific immunity, the majority of our adolescent patients showed robust humoral responses despite antimetabolite-based treatment being associated with poor vaccination outcomes in adults.

## Graphical Abstract

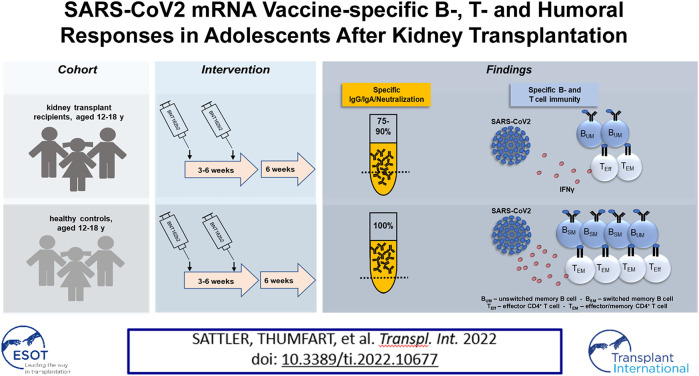

## Introduction

Children and adolescents frequently experience asymptomatic SARS-CoV-2 infections or exhibit mild respiratory symptoms with fever, headache, and cough. Nevertheless, both groups can suffer from severe COVID-19 with respiratory failure or pediatric inflammatory multisystem syndrome (1). Children on renal replacement therapy or after kidney transplantation (KTx) have an increased risk for hospitalization after infection (2). Even if mortality is low, pediatric COVID-19 can seriously burden health care systems in times of pandemic, as treatment resources may become scarce. It should also be noted that parents are particularly concerned about the health of their chronically ill children, resulting in higher rates of homeschooling, with adverse consequences for social interaction with peers (3). Vaccination against SARS-CoV-2, being recommended by the World health organization for children and adolescents with underlying chronic diseases (4), is therefore key for protection of pediatric at-risk groups. However, many studies (5–7) revealed that adult solid organ recipients show a broad impairment in CoV2-vaccination-induced immunity, affecting both humoral and cellular responses, likely due to mycophenolate (MPA)-based immunosuppression (IS) (8). To provide comprehensive data on mRNA vaccine induced responses in kidney transplant recipients (KTR) aged 12–18 years, we conducted an observational study after approval for this age group where vaccine-specific IgG, IgA and virus neutralizing capacity was assessed in concert with comprehensive quantification and functional characterization of spike protein-specific B- and T cells. Our results suggest that the majority of pediatric patients, despite being on antimetabolite treatment, mount robust vaccine-specific humoral responses, with selective impairments in various adaptive immune compartments.

## Materials and Methods

### Study Design and Medication

For this observational study, adolescent KTx patients were recruited at the Charité Department of Pediatric Gastroenterology, Nephrology and Metabolic Diseases. As per medical center guideline, patients had initially received triple IS therapy consisting of corticosteroids (CS), Tac and MPA immediately after KTx. Except for patient 1 (Table 2), none of the patients had received induction therapy. After the first year, CS had been discontinued according to the standard protocol of the center. Healthy controls included age- and sex-matched individuals without any documented acute or chronic disease conditions (Table 1). They were routinely vaccinated according to the national vaccination program. The inclusion criteria for the study groups were age between 12 and 18 years, absence of previous SARS-CoV2 or other severe infections prior to vaccination and completion of the 2-dose vaccination protocol with BNT162b2 vaccine (Comirnaty; BioNTech/Pfizer) according to the manufacturer’s recommended dose (30 µg) and time schedule (two doses at a 3–6 weeks interval). All patients received the 2nd dose between June and October 2021. Blood and serum samples were collected approximately 6 weeks after the second dose with no significant differences between groups. IS trough levels (Table 2) were analyzed at the same time point. The study was approved by the local ethical committee of the Charité Universitätsmedizin (EA2/227/21). All individuals (over 14 years) and their legal guardians signed informed consent.

**TABLE 1:** Basic characteristics of KTx patients and healthy controls.

Variable	KTx (*n* =20)	HC (*n* = 13)	*p value*
Age (mean yrs ±SD)	14.17 (1.31)	13.99 (1.99)	0.7595
Females (n, %)	7 (35.00)	5 (38.46)	>0.9999
Caucasians (n, %)	16 (80.00)	12 (92.00)	0.6253
Time since 2nd vaccination dose (mean days±SD)	39.30 (11.06)	44.54 (16.87)	0.2306

(K)Tx, (Kidney) Transplantation; HC, healthy control.

**TABLE 2:** Detailed characteristics of KTx patients.

Patient	Gender	Underlying disease	Age (years)	Time since KTx (years)	Current IS^b^	Tac trough level (µg/l)^b^	MPA trough level (mg/l)^§^	Former rejection episodes (date)
1^a^	F	unknown	17.5	0.9	Tac, MMF, CS	5.1	3.3	Yes (2021/2, 2021/3)
2	F	CAKUT	13.1	9.2	Tac, MMF	2.7	8.3	No
3	M	CAKUT	12.6	9.6	Tac, MMF	3.3	0.9	No
4	M	CAKUT	12.5	10.0	Tac, MMF	3.3	1.3	No
5	M	CAKUT	13.2	7.1	Tac, MMF	3.3	2.1	No
6	M	ARPKD	12.8	1.8	Tac, MMF	4.8	1.8	No
7	M	CAKUT	14.9	13.2	Tac, MMF	4.2	3.5	No
8	M	CAKUT	15.0	8.3	Tac, MMF	3.6	0.8	No
9	F	NS	14.6	10.2	Tac, MMF	5	2.5	No
10	F	NS	15.1	10.2	Tac, CS	3.3	n.a	No
11	F	NS	14.1	2.6	Tac, Eve	5.7	n.a	Yes (2019/4)
12	M	HNF1 Beta	14.9	2.1	Tac, MMF	4.7	1.2	No
13	M	NS	16.1	10.6	Tac, CS	4.9	n.a	No
14	F	Papillorenal syndrome	13.1	1.0	Tac, MMF	3.6	4.3	No
15	M	NS	15.3	7.4	Rapa, MMF, CS		4.5	No
16	M	Sartan nephropathy	14.2	10.5	Tac, CS	2.8	n.a	No
17	M	CAKUT	14.0	11.7	Tac, MMF	4.1	0.4	Yes (2012/10)
18	F	NS	14.3	9.2	Tac, MMF, CS	5.7	1.7	Yes (2014/9, 2014/12)
19	M	Cystinosis	12.6	2.1	Tac, Eve	1.8	n.a	No
20	M	CAKUT	13.4	3.3	Tac, MMF	3.7	1.8	No
Mean ± SD			14.2 ± 1.3	7.0 ± 4.1		4.0 ± 1.1	1.8 ± 2.0	

(K)Tx, (Kidney) Transplantation; IS, immunosuppression; Tac-Tacrolimus; MPA-Mycophenolic Acid; CS-Corticosteroids; Eve-Everolimus; Rapa-Rapamycin; CAKUT, congenital anomalies of kidney and urinary tract; NS, nephrotic syndrome; ARPKD-Autosomal recessive polycystic kidney disease; SD, standard deviation; n.a., not applicable.

a Patient 1 had received her 2nd transplant with induction therapy (basiliximab).

b At time of humoral and cellular analysis.

### Assessment of Humoral Immunity

Previous or current SARS-CoV2 infection was excluded based on medical history data available from our clinic, continuous negative point of care antigen tests conducted thrice-weekly during school visits and a negative SARS-CoV2 nucleoprotein specific ELISA (Euroimmun) (Supplementary Figure S1A). SARS-CoV-2 S1 domain specific IgG (QuantiVac, Euroimmun) and IgA (Euroimmun) was determined by ELISA. For IgA quantification, serum samples exceeding O.D. ratios of 6 were pre-diluted tenfold and re-measured. Serum samples with OD ratios of ≥1.1 (Nucleoprotein and IgA) or ≥35.2 BAU/ml (IgG) were considered positive according to the manufacturer´s guidelines. OD ratios were calculated based on the ratio of the OD of the respective sample over the OD of the calibrator provided with the ELISA kit. For quantification of virus neutralizing capacity in our study, a blocking ELISA (sVNT kit, GenScript) was used that mimics the virus neutralization process. In detail, serum antibodies are tested for blocking the binding of recombinant SARS-CoV2 RBD (alpha = vaccine variant) to human ACE2 receptor protein. A neutralization capacity of more than 30% was defined as positive as per the manufacturer’s recommendation.

### Assessment of SARS-CoV2 Vaccine-Specific B and T Cells

Peripheral blood mononuclear cells (PBMCs) were isolated from EDTA blood by density gradient centrifugation using Ficoll-Paque PLUS (GE Healthcare Bio-Sciences, Chicago, IL, United States). Within 5–10 × 10^6^ PBMC, B cells were detected by flow cytometry and gated as CD19^+^CD3^−^CD14^−^CD56^−^ among single live lymphocytes (gating strategy depicted in Supplementary Figure S1B). SARS-CoV2-specific B cells were identified as shown before (8, 9) by double staining with AlexaFluor488 coupled recombinant receptor binding domain (RBD) protein and biotinylated recombinant full spike protein (both alpha-variant, RnD Systems, Minneapolis, MN, United States) with the latter being detected by streptavidin-APC (Biolegend, San Diego, CA, United States). For flow cytometric analysis, the following fluorochrome-labeled antibodies were used: CD19 (SJ25C1, BL), CD3 (SK7, BL), CD56 (NCAM, BL), CD14 (M5E2, BL), IgD (IA6-2, BL), IgG (G18-145, BD) and CD27 (M-T271, BL). For identification of vaccine-specific T cells, 3 × 10^6^ PBMC were stimulated or not for 16 h with overlapping 15-mers covering the complete SARS-CoV2 spike (alpha-variant) protein. A combination of overlapping 15-mer peptide mixes including cytomegalovirus (CMV, “Peptivator pp65,” Miltenyi Biotech, Bergisch Gladbach), Epstein Barr virus (EBV, “Peptivator consensus”, Miltenyi Biotech) and influenza H1N1 (“Peptivator matrix protein 1,” and “Peptivator nucleoprotein,” Miltenyi Biotech) served as control and is termed CEF throughout. Antigens were used at a final concentration of 0.5 μg/ml per peptide. Specific CD4^+^ T helper cells were identified based on CD137 and CD154 coexpression as shown in Supplementary Figure S2. A response was defined as positive when stimulated cultures contained at least twofold higher frequencies of CD137^+^CD154^+^ cells as compared to the respective unstimulated control with at least twenty events, as resported earlier (5). For surface labelling, antibodies against CD3 (SK7, Biolegend), CD4 (SK3, BD), CD8 (SK1, Ebioscience, San Diego, CA, United States), CD45RO (UCHL1, BL), CD62L (DREG-56, BL) and PD1 (EH12.1, BD) were used. A dump channel excluded unwanted cells and contained CD14^+^ (M5E2, BL), CD19^+^ (HIB19, BL), and dead (fixable live/dead, BL) events. Cells were fixed with FACS Lysing Solution (BD) after surface staining, followed by permeabilization in FACS Perm II Solution (BD) and stained intracellularly with anti-CD154 (24–31, BL), anti-CD137 (4B4-1, BL), anti-TNF-α (MAb11, BL), anti-IFN-γ (4SB3, Ebioscience), anti-IL-2 (MQ1-17H12, BL), and anti-IL-4 (MP4-25D2, BL). Data was acquired using a BD FACS Fortessa X20.

### Data Analysis and Statistics

FACS data analysis was conducted with FlowJo 10 (BD). Gating strategies for analysis of antigen-reactive B- and T cells are illustrated in Supplementary Figures S1, S2. Depicted frequencies of spike-specific CD4^+^ T cells were background (=unstimulated control) -substracted. Co-expression of cytokines was quantified by Boolean gating in Flowjo. Statistical analysis and graph preparation was performed in GraphPad Prism 8 (GraphPad, La Jolla, CA, United States). Data distribution was assessed using the Kolmogorov-Smirnov test. Given that all data sets did not show normal distribution, a Mann-Whitney test was used throughout for two-group comparisons. For analysis of contingency tables, Fisher’s exact test was applied.

## Results

### Patient Characteristics

Twelve patients received dual IS therapy with Tac and MPA according to the standard protocol of the center after the first year after KTx. Two patients received triple IS therapy (Tac, MPA and CS) due to former rejection episodes. Three patients received Tac and CS due to side effects of MPA. Two patients were treated with Everolimus and Tac because of ongoing Epstein-Barr- and polyoma virus BK viremia. One patient received triple IS (Rapamycin, MPA and corticosteroids) due to calcineurin inhibitor toxicity. Mean trough levels were 4.0 ± 1.1 for Tac and 1.8 ± 2.0 for MPA at the time point of immunity analysis (Table 2); no changes in medication were undertaken from 2 months before vaccination until time point of humoral and cellular analyses. Last rejection episodes with methylprednisolone treatment were at least 4 months before first vaccination. After vaccination, no rejection episodes were observed in the KTx cohort. No other adjunctive immunosuppressive drugs were taken within the last 6 months prior to analysis. As per inclusion criteria, all patients and controls were virus-naïve at the timepoint of analyses and none of the individuals became SARS-CoV2-positive until the end of the study (12/2021).

### Characterization of SARS-CoV2-Vaccination-Specific Humoral and B Cell Immunity

**FIGURE 1:**
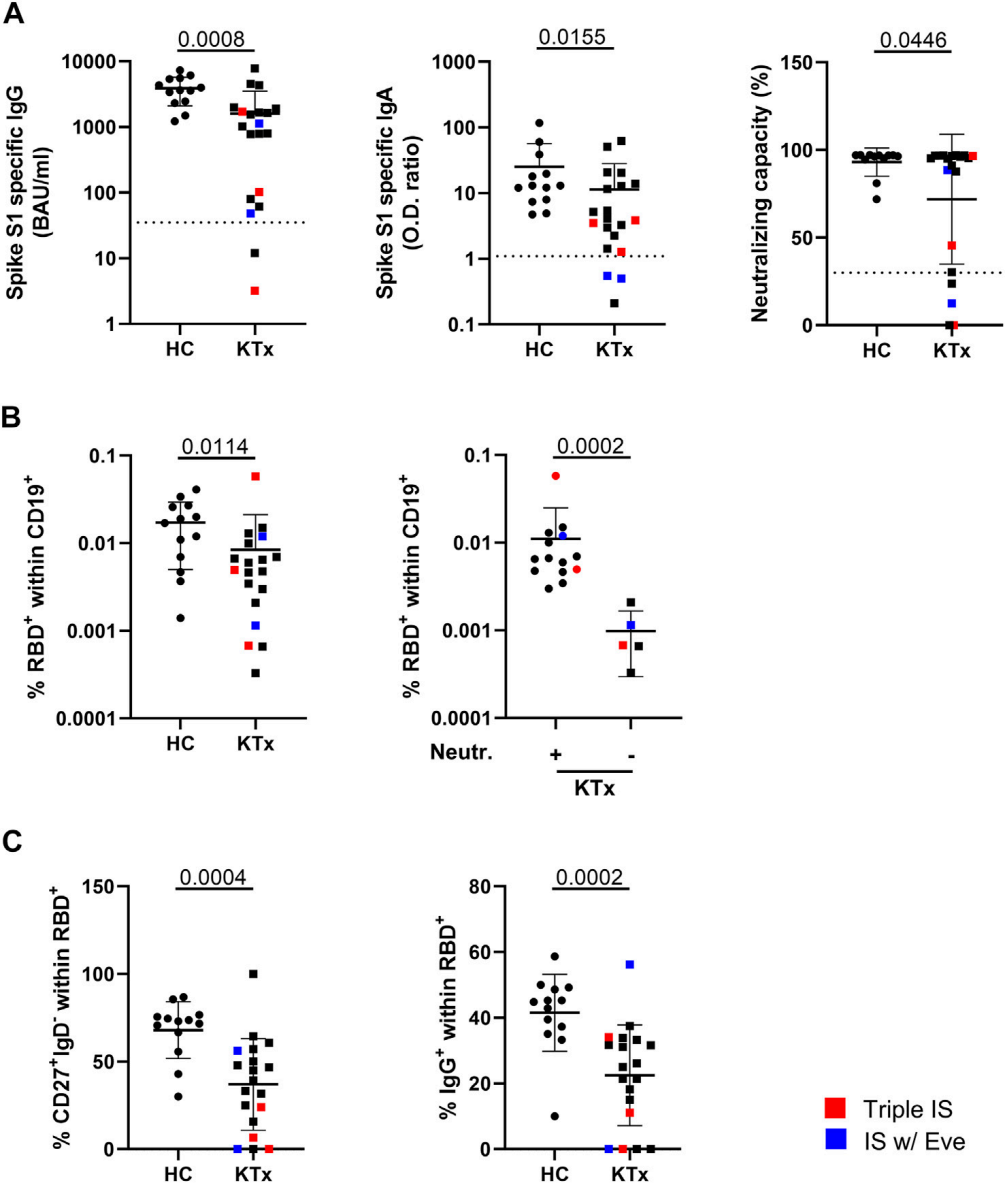
Humoral immune responses and vaccination-specific B cell immunity. **(A)** Humoral vaccine-specific immune responses were assessed for anti-spike protein S1 domain specific IgG, IgA and virus neutralization capacity in healthy controls (HC) and KTx patients around 6 weeks after administration of the second standard BNT162b2 dose. Thresholds defining a positive response are indicated by dotted lines. **(B)** Relative frequencies of RBD-specific CD19^+^ B cells identified by FACS in HC vs KTx (left) and in KTx that were stratified according to responders (+) and non-responders (−) regarding neutralizing capacity above threshold (right). **(C)** Memory-type of spike-RBD-specific B-cells according to IgD and CD27 expression (left) and portions of class-switched IgG^+^ cells (right). In all analyses, *n* = 20 KTx and *n* = 13 HC were enrolled. Mann-Whitney test was applied throughout based on non-normal distribution of values. Graphs show means ± SD. Patients receiving triple immunosuppressive (IS) therapy are depicted in red, those under Everolimus-containing IS in blue.

Humoral BNT162b2-vaccination-specific immunity above threshold was detected in all healthy individuals and in 90% (18/20) of KTR for IgG, 85% (17/20) for IgA and in 75% (15/20) with respect to neutralizing capacity. Two of three patients receiving triple IS therapy showed no or low IgG and neutralizing antibody levels. One of the two patients under Everolimus treatment showed low IgG and no neutralizing antibody titers whereas both did not develop IgA responses. Overall, KTx patients showed significantly reduced spike-specific IgG, IgA- and neutralization capacity levels as compared to controls (Figure 1A). Employing a robust FACS-based assay (9), transplant recipients were further characterized by significantly reduced frequencies of spike protein receptor binding domain (RBD)-specific B cells (Figure 1B, left) that were approximately ten-fold higher in humoral responders as compared to non-responders (Figure 1B, right). Antigen-specific B cells in patients contained reduced portions of isotype class switched IgD^−^CD27^+^ memory-type cells (Figure 1C, left), being in line with diminished frequencies of specific IgG^+^ cells (Figure 1C, right). An exemplary gating strategy for B cell characterization is depicted in Supplementary Figure S1.

### Quantitative and Qualitative Features of Vaccination-Specific CD4^+^ T Cells

**FIGURE 2:**
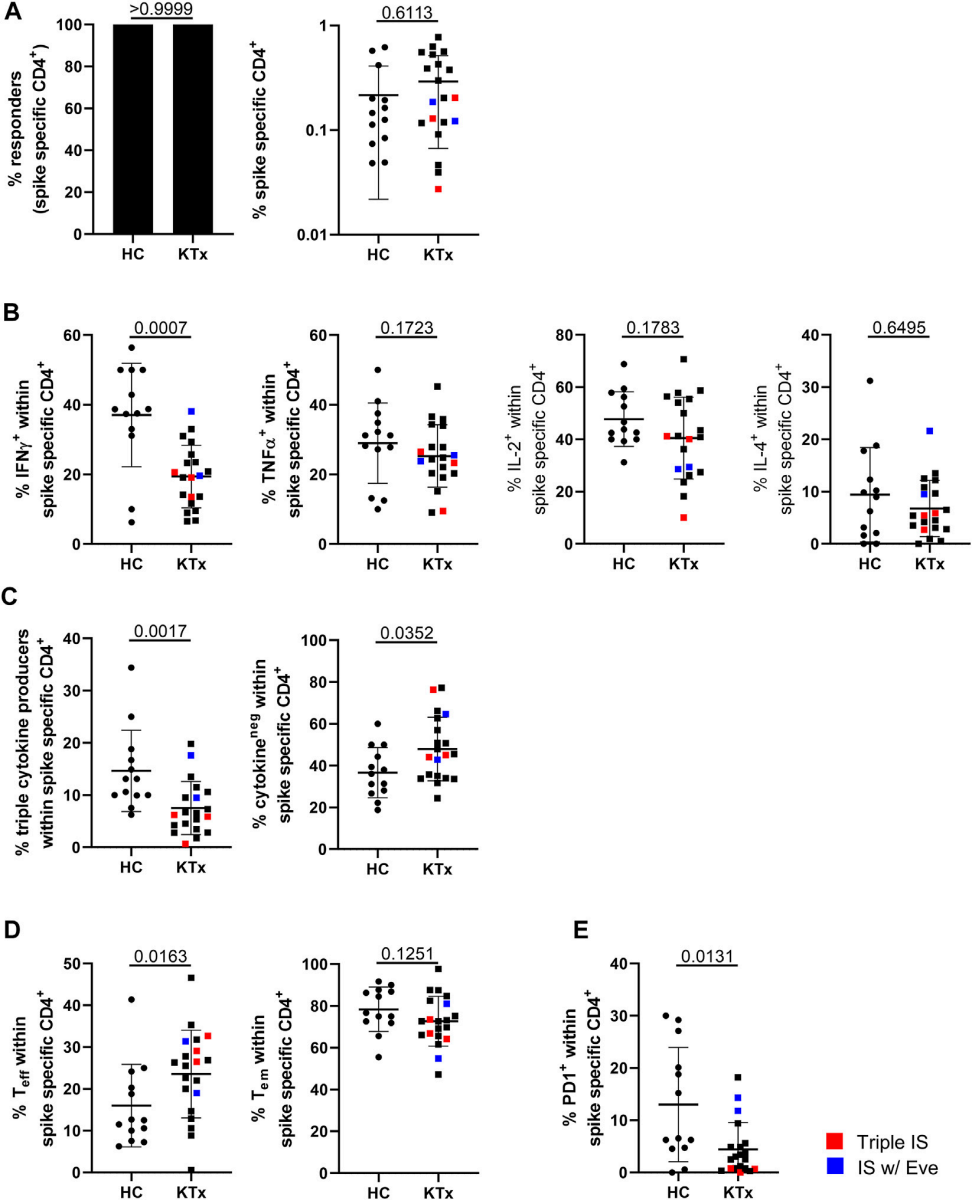
Assessment of spike-specific T cell responses. PBMC were stimulated with spike peptide mix or left unstimulated. Specific CD4^+^ T cells were detected by flow cytometry according to co-expression of CD154 and CD137. **(A)** Depicts responder rates (left) and portions of spike-specific CD4^+^ T cells in HC vs. KTx after unstimulated control background substraction (right), **(B)** frequencies of cytokine producers as indicated within the antigen-specific population. **(C)** Quantifies IFNγ^+^TNFα^+^IL-2^+^ “triple^+^” polyfunctional (left) and cytokine-non-producing (right) cells. **(D)** Analysis of effector and effector/memory-type differentiation of antigen-specific CD4^+^ T cells. **(E)** Portions of specific, recently *in vivo* activated PD-1^+^ T cells. N in all experiments as in Figure 1. Mann-Whitney test was applied throughout based on non-normal distribution of values. Graphs show means ± SD. Patients receiving triple immunosuppressive therapy are depicted in red, those under Everolimus-containing IS in blue.

Vaccination-specific SARS-CoV2 spike protein- and CEF control antigen-reactive CD4^+^ T cells were identified based on CD154 and CD137 coexpression after peptide mix stimulation with the gating strategy illustrated in Supplementary Figure S2. We detected spike-specific CD4^+^ T cells in all individuals included in the study (Figure 2A, left) with frequencies being similar in patients and controls (Figure 2A, right). However, KTx patients were characterized by significantly reduced portions of IFNγ^+^-, but not TNFα^+^, IL-2^+^ or IL-4^+^ T cells (Figure 2B). Furthermore, antigen-reactive polyfunctional T cells co-expressing IFNγ, TNFα and IL-2 were less frequently detected in transplanted individuals, along with higher frequencies of cytokine non-producing cells (Figure 2C). IL-4 was excluded from polyfunctionality analyses due to comparably low frequencies of positive cells.

With respect to subset classification of antigen-specific cells, we found a significant increase of CD45RO^−^CD62L^−^ effector T cells in patients in concert with lower portions of CD45RO^+^CD62L^−^ effector/memory-type T cells (Figure 2D). Furthermore, healthy donors showed significantly elevated portions of antigen-specific PD1^+^ cells than KTx, reflecting recent *in vivo* activation (Figure 2E). Overall frequencies of antigen-specific T cells were within the lower range in one patient, and *in vivo* activated PD-1^+^ cells were rarely found in all three patients under triple IS treatment. Of note, CEF control antigen mix specific CD4^+^ T cell responses did not significantly differ between healthy controls and patients except for slightly reduced frequencies of IL-2^+^ T cells in the latter (Supplementary Figures S3A–E).

## Discussion

A plethora of studies suggests that vaccination of adult KTx patients against SARS-CoV2 results in blunted antiviral immunity (6, 7), mirrored by the broad inability to develop neutralizing antibody titers in individuals receiving standard triple IS including antimetabolites (5, 9). So far, reactogenicity of mRNA vaccines in pediatric patients has only been examined for individuals with a mean age of 18 years and only with respect to IgG responses in the absence of matched healthy controls (10, 11). Importantly, our data presented herein demonstrate that 85–90% of our KTx patient cohort between 12 and 18 years of age developed IgA and IgG responses, respectively, while 75% reached neutralizing antibody titers. According to recent literature, the latter data based on sVNT assay measurement might potentially even underestimate neutralizing capacity as compared to the Plaque Reduction Neutralization Test (12).

Whereas these results are encouraging and suggest that high humoral responder rates can be achieved despite MPA treatment, our data at the same time reveal that all arms of adaptive immunity are compromised in young patients as compared to controls. This includes frequencies of spike-reactive B cells and their capacity to undergo class switching to IgG, a phenomenon already reported for adult cohorts (9) and likely resulting from Everolimus- (13) or MPA-based suppression of B cell differentiation and plasma blast formation (14, 15). In fact, we could recently show that short-term pausing of MPA during SARS-CoV2 re-vaccination enables previous non-responders to mount robust anti-viral immunity including expansion of antigen-specific B cells (8). The absence of antimetabolites also supported specific T cell proliferation and *ex vivo* activation, whereas cytokine production capacity was only marginally affected (8). Interestingly, pediatric KTx patients showed selective limitations within spike-specific T cells as compared to controls that mainly included memory differentiation, IFNγ production and polyfunctionality. Whereas the exact role of multifunctional T cells is not completely understood, they might contribute to a better protection given that quantities are elevated in individuals experiencing mild as compared to severe SARS-COV2 infections (16), a feature also observed in other infections such as tuberculosis (17).

With respect to cytokine production, adolescent KTx patients obviously show less impairment than their adult counterparts where production of all spike- induced, but not CEF-induced cytokines, was strongly blunted (5). As one limitation of our study, it remains to be determined whether these differences predominantly depend on patient age, as has been already discussed for HBV vaccination in transplant recipients (18) or arise from different treatment regimens, given that the default medication recommendation of adult transplant recipients comprises triple IS including corticosteroids, whereas 60% of our pediatric patients received dual IS with Tac and MPA. In support of the latter hypothesis, two of three adolescents under triple IS in our study showed no or low specific IgG levels; the same applied to frequencies of class-switched memory B-cells. Given the potential risk of rejection episodes, however, therapeutic modifications including MPA hold are probably not reasonable in pediatric KTx patients.

The main limitation of our study is the relatively small study cohort. However, due to ethical guidelines limiting blood donation volumes from young individuals for cellular assays and a high dissemination of CoV2-infection in this group (thereby preventing inclusion of more virus-naive individuals), studies on adolescents will likely remain comparably small. Additionally, the overall number of adolescent KTx patients is substantially lower and vaccine approval was delayed as compared to adults. These facts may explain the comparably small size of other pediatric studies (10, 11). Due to the completion of our study by the end of 2021, we were not able to include data after a third vaccination of our cohort that is meanwhile standard of care. Given that recent literature demonstrates a considerable impact of a booster immunization on IgG levels in adolescent transplant recipients (19), it will not only be important to examine all arms of immunity after a third dose to better understand differential vaccine-specific immunity of young vs adult KTx patients, but also include neutralization data on virus variants of concern that have emerged meanwhile and have not been considered in this study.

In summary, based on comprehensive SARS-CoV2 vaccine-specific serological and cellular analysis, our data demonstrate that the majority of pediatric KTx patients under dual IS therapy in our cohort develops robust humoral immunity, but shows distinct differentiation- and function-related impairments within B- and T helper cell compartments.

## Data Availability

The raw data supporting the conclusion of this article will be made available by the authors, without undue reservation.

## Abbreviations

ARPKD, autosomal recessive polycystic kidney disease; CAKUT, congenital anomalies of kidney and urinary tract; CS, corticosteroids; Eve, everolimus; HC, healthy control(s); IS, immunosuppression; KTx, kidney transplantation; KTR, kidney transplant recipients; MPA, mycophenolic Acid; NS, nephrotic syndrome; PBMC, peripheral blood mononuclear cells; RBD, receptor binding domain; Rapa, rapamycin; Tac, tacrolimus.
