# Supplementary material for: SARS-CoV2 mRNA Vaccine-Specific B-, T- and Humoral Responses in Adolescents After Kidney Transplantation
Source: Transpl Int. 2022 Aug 4;35:10677. doi: 10.3389/ti.2022.10677 (PMC9385879; doi:10.3389/ti.2022.10677)
Supplement: Supplementary file 1 [file DataSheet1.pdf]

Supplemental Figure 1 Sattler, Thumfart et al.

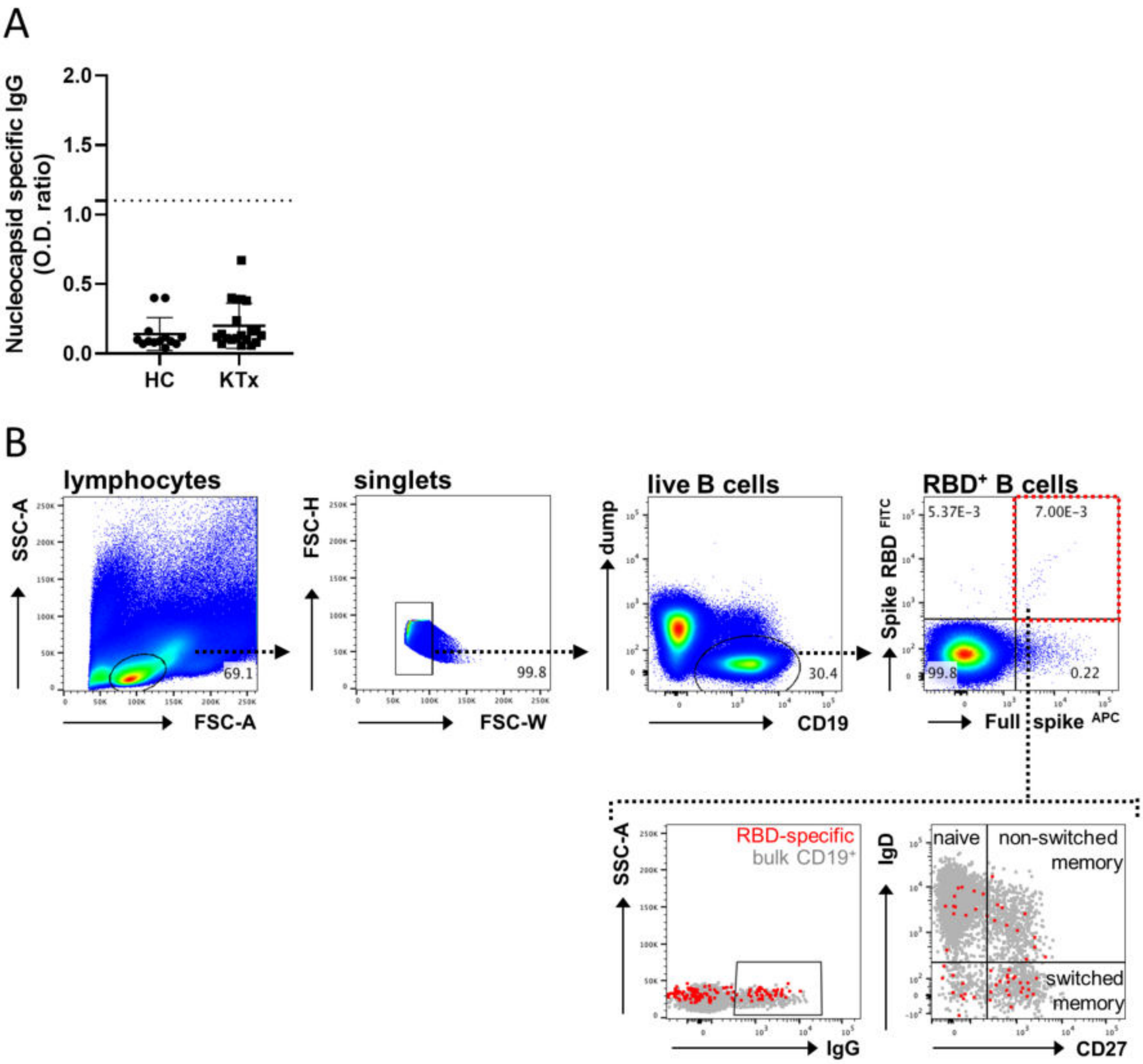

Exclusion of previous SARS-CoV2-infection and detection and characterization of SARS-CoV-2 spike RBD specific B cells. (A) Detection of viral nucleocapsid-protein specific antibodies by ELISA with the cut-off for positivity at an O.D. ratio of 1.1 (dotted line). (B) Antigen-specific live single CD14^{-}CD56^{-}CD3^{-} ("dump" negative) CD19^{+} B cells were identified by flow cytometry based on co-staining with recombinant spike RBD-FITC and recombinant full spike-APC. Specific cells were further analyzed for memory differentiation (CD27, IgD) and IgG expression.

Supplemental Figure 2 Sattler, Thumfart et al.

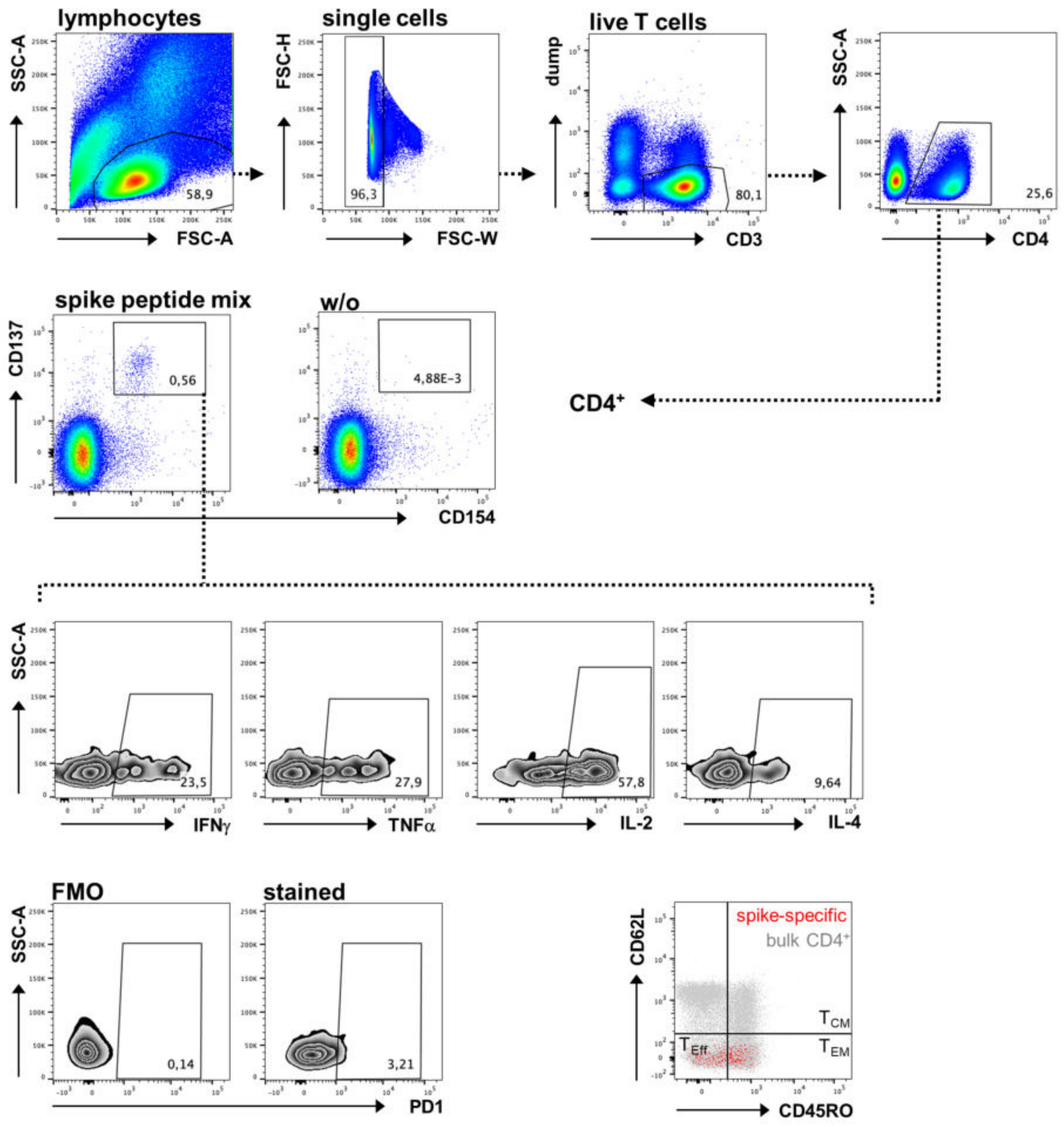

FACS-based identification and characterization of SARS-CoV2 vaccine specific $CD4^+$ T cells. PBMC were stimulated or not for 16 h with SARS-CoV2 spike protein peptide mix. Antigen-specific live single $CD14^-$ $CD19^-CD3^+$ ("dump" negative) $CD4^+$ T cells were identified by flow cytometry based on co-expression of CD154 and CD137. Specific cells were further analyzed for expression of cytokines (IFNγ, TNFα, IL-2 and IL-4), for the in vivo induced activation related marker PD1, or for their memory differentiation phenotype based on CD45RO/CD62L expression (T_{CM}: central memory-, T_{EM}: effector memory-, T_{eff}: effector T cells). Gates were set according to the respective unstimulated or unstained ("fluorescence minus one" - FMO) controls.

Supplemental Figure 3 Sattler, Thumfart et al.

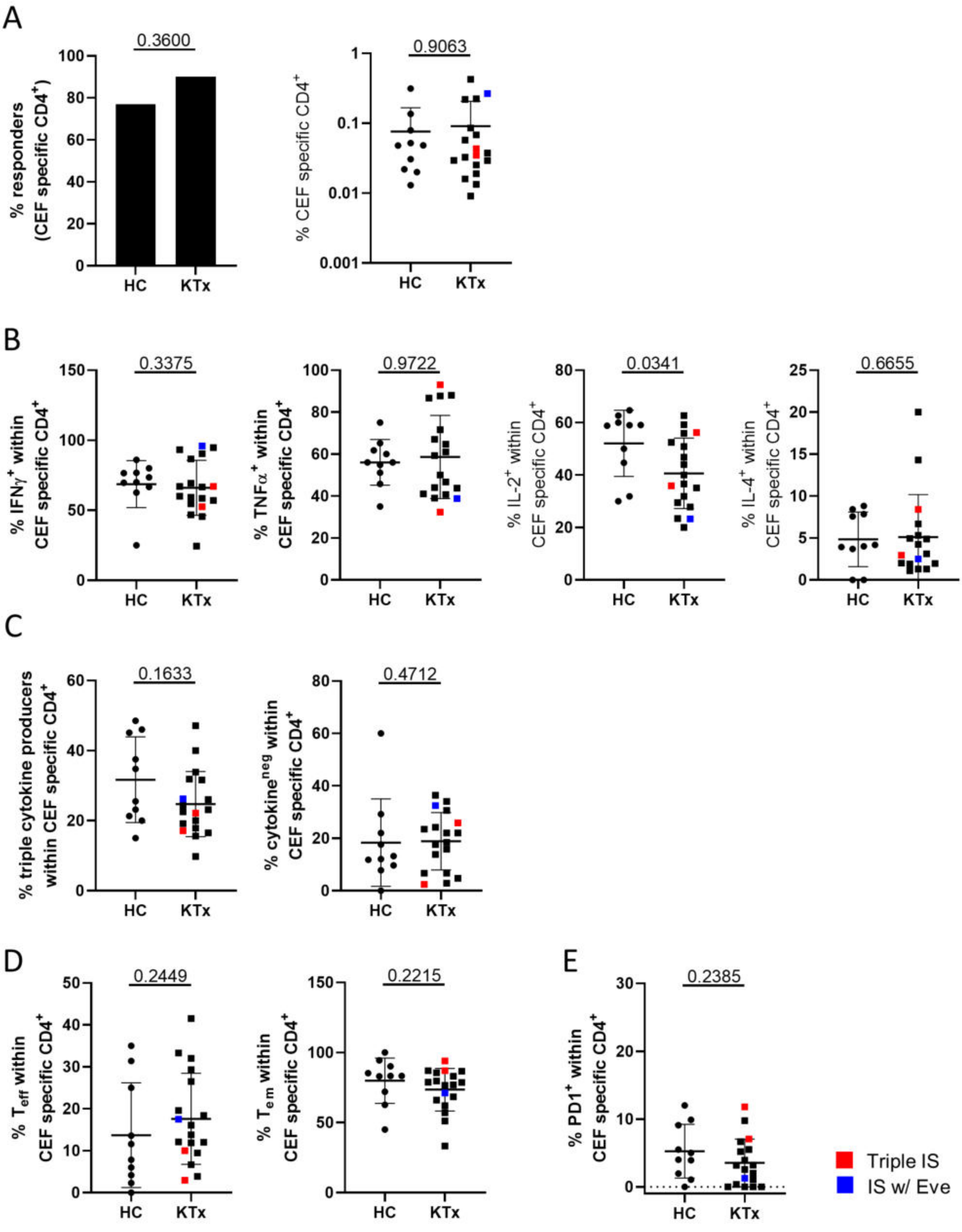

Characterization of CEF-specific CD4+ T cells. PBMC were stimulated or not for 16 h with CEF peptide mix. (A) depicts overall responder rates (left) and frequencies after background substraction (right), (B) frequencies of cytokine positive and (C) polyfunctional (IFNγ+TNFα+IL-2+, left) or cytokine negative (right) cells. (D) quantifies memory or effector subsets, while (E) depicts frequencies of PD-1+ CEF-specific T cells.
